# Perceived knowledge of psychiatry and family medicine residents regarding medical management of schizophrenia, hypertension, diabetes mellitus, and dyslipidemia: opportunities to refine the residency training

**DOI:** 10.1186/s12909-021-02658-z

**Published:** 2021-04-22

**Authors:** Jiangbo Ying, Jinhui Wan, Kang Sim, Ee-Jin Darren Seah, Mythily Subramaniam

**Affiliations:** 1 National Psychiatry Residency Program, National Healthcare Group, Singapore, Singapore; 2 Family Medicine Residency Program, National Healthcare Group, Singapore, Singapore; 3 National Healthcare Group Polyclinics, Singapore, Singapore; 4 West Region, Institute of Mental Health, Singapore, Singapore; 5 Research Division, Institute of Mental Health, Singapore, Singapore

**Keywords:** Psychiatry resident, Family medicine resident, Schizophrenia, Metabolic syndrome, Knowledge

## Abstract

**Background:**

Psychiatry and Family Medicine residents frequently see patients with comorbid mental and physical disorders. Little is known about the difference in knowledge of Psychiatry residents and Family Medicine residents regarding management of common conditions they encounter. This study aimed to assess the knowledge of Psychiatry and Family Medicine residents regarding medical management of schizophrenia, hypertension, diabetes mellitus, and dyslipidemia, as the findings could help to refine the training curriculum for residency training.

**Methods:**

A cross-sectional survey design was used. Psychiatry and Family Medicine residents pursuing their residency in Singapore were recruited from November 2019 to June 2020. The survey questionnaire consisted of questions which assessed the knowledge regarding medical management of schizophrenia, hypertension, diabetes mellitus, and dyslipidemia. Descriptive statistics were used to describe the demographic data; T-tests or Mann-Whitney U tests to compare the differences between groups; and multiple regression analyses to assess the factors associated with Psychiatry residents’ knowledge of hypertension, diabetes mellitus, and dyslipidemia.

**Results:**

Fifty-seven out of 70 (81.4%) Psychiatry residents and 58 out of 61 (95.1%) Family Medicine residents participated in the study. The majority of Psychiatry residents encountered patients with hypertension (93.0%), diabetes mellitus (87.7%) and dyslipidemia (91.2%) on a daily to weekly basis. Psychiatry residents had higher scores on questions about schizophrenia versus Family Medicine residents (mean 50.70 versus 43.28, *p* < 0.001). However, Psychiatry residents scored lower on questions about hypertension (mean 33.86 versus 40.98, *p* < 0.001), diabetes mellitus (mean 45.68 versus 49.79, *p* = 0.005) and dyslipidemia (mean 37.04 versus 44.31, *p* < 0.001). Receiving undergraduate medical education locally, compared to receiving it overseas, was associated with better knowledge of hypertension (beta = 0.515, *p* = 0.009) and dyslipidemia (beta = 0.559, *p* = 0.005); while younger age (26–30 versus > 35 and 31–35 versus > 35) was associated with better knowledge of hypertension (beta = 1.361, *p* = 0.002 and beta = 1.225, *p* = 0.003). A significant proportion of Psychiatry residents (61.4%) did not agree that the training provided to manage hypertension, diabetes mellitus, and dyslipidemia was adequate. Similarly, majority of Family Medicine residents (62.1%) did not agree that they had adequate training to manage schizophrenia.

**Conclusions:**

This study raises the awareness of Psychiatry residents’ sense of discomfort in managing hypertension, diabetes mellitus, or dyslipidemia and conversely Family Medicine residents in management of schizophrenia, which can be further addressed during the training postings within the residency programs. Future studies are needed to look at local (such as training curriculum) and systemic factors (such as practice trends and culture) in order to better align residency selection criteria and training foci with real world practice factors over time.

**Supplementary Information:**

The online version contains supplementary material available at 10.1186/s12909-021-02658-z.

## Background

Recently there is increased awareness of the importance of collaboration between Psychiatry and Family Medicine both in Singapore and other countries [1–4]. Both Psychiatry residents and Family Medicine residents frequently see patients with comorbid mental and physical disorders, but present management of these patients does not take an integrated approach in Singapore. Psychiatrists focus on mental disorders and leave the treatment of physical disorders to their medical colleagues, and vice versa. There appears to be a sense of discomfort in managing illnesses outside one’s specialty, with increasing referrals between different specialties [5].

Schizophrenia is a severe and chronic mental disorder. Globally it was ranked among the top 15 leading causes of disease-related disability in 2016 [6]. Patients with schizophrenia have an increased risk of metabolic syndrome, a constellation of clinical features, including hypertension, diabetes mellitus, and/or dyslipidemia [7]. Metabolic syndrome is associated with an increased risk of cardiovascular diseases and increased all-cause mortality rate [8]. A recent meta-analysis reported that the prevalence of metabolic syndrome is 58% higher in psychiatric patients compared with the general population [9]. In Singapore, the prevalence of metabolic syndrome in patients with schizophrenia was reported to be 46.0% [10].

Due to the high prevalence of metabolic syndrome in patients with schizophrenia, it is important to consider integrated management of schizophrenia, hypertension, diabetes mellitus, and dyslipidemia. Collaborative management can improve health maintenance and patient satisfaction and reduce health care costs [11, 12]. Psychiatry residents are trained to manage schizophrenia, but as reported in the extant literature [13], Psychiatry residents would just refer those patients with hypertension, diabetes mellitus, and/or dyslipidemia to family physicians. On the other hand, Family Medicine residents are familiar with the management of hypertension, diabetes mellitus, and dyslipidemia. However, one study revealed that Family Medicine residents had low comfort levels in seeing patients with mental illness [14]. Whilst Psychiatry residents may not manage complex cases of hypertension, diabetes mellitus, or dyslipidemia, better health care services can be provided if Psychiatry residents have adequate knowledge in managing these conditions in their early stages or work collaboratively with colleagues in Family Medicine. Clinical knowledge is one of the important components of clinical confidence [15]. Research has revealed that lack of knowledge may have a significant impact on patient care [16]. It is essential to find out whether Psychiatry residents have a certain level of knowledge regarding the medical management of hypertension, diabetes mellitus, and dyslipidemia, as we are considering the integrated management of schizophrenia and these conditions.

Since 2010, Singapore has adopted a residency system for postgraduate medical education under the purview of the Accreditation Council for Graduate Medical Education-International (ACGME-I) [17]. The National Healthcare Group (NHG) National Psychiatry Residency Program is a 5-year training program to train Psychiatry residents in the management of a wide range of psychiatric conditions to meet the country’s mental health needs and the promotion of psychological well-being [18]. The current curriculum of NHG National Psychiatry Residency Program comprises of postings in Medicine and Neurology in which psychiatry residents see and manage patients with hypertension, diabetes mellitus, and dyslipidemia. The NHG Family Medicine Residency Program is a 3-year broad-based training program and aims to equip Family Medicine residents with the skills and knowledge to provide comprehensive inter-generational care to patients [19]. Family Medicine residents will see patients with medical disorders in polyclinics throughout their 3-year residency program. They are also expected to manage patients with stable mental disorders, such as patients with chronic schizophrenia.

This study aimed to assess the knowledge of Psychiatry residents and Family Medicine residents regarding medical management of schizophrenia, hypertension, diabetes mellitus, and dyslipidemia. This study specifically focused on evaluating whether Psychiatry residents, when compared with Family Medicine residents, have adequate medical knowledge of managing hypertension, diabetes mellitus, and dyslipidemia, as the findings could help to refine the training curriculum for residency training.

## Methods

### Study participants

A cross-sectional survey design was used in this study. Psychiatry residents in the NHG National Psychiatry Residency Program and Family Medicine residents in the NHG Family Medicine Residency Program in Singapore were recruited from November 2019 to June 2020. All 70 Psychiatry residents (first year residents to fifth year residents), and all 61 Family Medicine residents (first year residents to third residents) were invited to participate in this study face to face during their regular residency program tutorials (before the outbreak of Coronavirus Disease 2019 or COVID-19) or via emails (after the outbreak of COVID-19). Details of the study were explained to the participants face to face (before the outbreak of COVID-19) or via the video conferencing tool Zoom (after the outbreak of COVID-19). Signed informed consent was obtained before the administration of the anonymous questionnaires. A S$30 grocery voucher was given to each participant as an inconvenience fee on completion of the survey. When a Psychiatry resident or a Family Medicine resident declined to participate in the study, they were not asked to state the reasons. This study was approved by the National Healthcare Group, Doman Specific Review Board, Singapore (Institutional Ethics Committee).

### Survey instrument

The survey questionnaire consisted of three parts (Additional file 1). Part I collected the demographic data, including age, gender, previous medical education and working experience, and year of residency. Part II assessed the knowledge regarding the medical management of schizophrenia, hypertension, diabetes mellitus, and dyslipidemia. Part III evaluated the overall confidence level of managing these conditions.

Part II of the questionnaire included statements on the medical management of schizophrenia, hypertension, diabetes mellitus, and dyslipidemia. These statements were extracted or modified from the Singapore Ministry of Health Clinical Practice Guidelines [20–23]. The statements were further modified after discussion with senior clinicians who were domain experts. The statements focused on the common medical treatment plans of these conditions, such as “oral antipsychotic should be used as first-line treatment for patients with an acute relapse of schizophrenia”, “use beta-blockers with caution in patients at risk of developing diabetes mellitus, as it raises blood glucose concentrations”, “metformin is usually contraindicated in the presence of severe renal or hepatic insufficiency” and “statins are the first line drug for both hypercholesterolemia and mixed hyperlipidemia when pharmacotherapy is indicated”.

Twelve statements were employed to assess the knowledge of each condition. Residents were asked to rate themselves after reading the statements on a Likert scale ranging from: 0 (don’t know), 1 (strongly disagree), 2 (disagree), 3 (neutral), 4 (agree), 5 (strongly agree). The total score for each condition was 60 (5 points × 12 questions). The scores for negatively worded questions were reversed for the analysis; a high score thus indicated better knowledge of the participant.

Part III of the questionnaire assessed the overall confidence level in managing patients with schizophrenia, hypertension, diabetes mellitus, and/or dyslipidemia using a six-level Likert scale ranging from 0 to 5, with a higher score indicating a higher confidence level.

### Statistical analysis

Descriptive statistics were used to report demographic data and frequency distribution. Shapiro-Wilk test was performed to check the normality of the data. Independent samples T-tests or Mann-Whitney U tests were performed to compare the differences between the Psychiatry and Family Medicine resident groups. Multiple regression analyses were used to assess the factors associated with Psychiatry residents’ knowledge of managing hypertension, diabetes mellitus, and dyslipidemia. All analyses were performed using IBM SPSS Version 25.

## Results

### Characteristics of study participants

Overall, 57 out of 70 (81.4%) Psychiatry residents and 58 out of 61 (95.1%) Family Medicine residents participated in the study. There were 26 males among the Psychiatry residents (45.6%) and 26 males among the Family Medicine residents (44.8%). Most participants were 26 to 30 years old. Most Psychiatry residents had up to 24 months of working experience in psychiatry (91.2%), but had no working experience in Family Medicine (96.5%) before they entered the psychiatry residency program. The majority of Psychiatry residents encountered patients with schizophrenia on a daily to weekly basis (98.2% of Psychiatry residents), and also encountered patients with hypertension (93.0%), diabetes mellitus (87.7%) and dyslipidemia (91.2%) on a daily to weekly basis. Nearly half of Family Medicine residents (48.3%) had provided consultations to patients with schizophrenia on a monthly basis. Table 1 summarizes the characteristics of study participants.

**Table 1:** Characteristics of study participants

Variables, n (%)		Psychiatry residents (***n*** = 57)	Family Medicine residents (***n*** = 58)
Gender	Male	26 (45.6)	26 (44.8)
	Female	31 (54.4)	32 (55.2)
Age, years	20–25	0 (0)	3 (5.2)
	26–30	32 (56.1)	51 (87.9)
	31–35	22 (38.6)	4 (6.9)
	> 35	3 (5.3)	0 (0)
Medical school	Local	25 (43.9)	36 (62.1)
	Overseas	32 (56.1)	22 (37.9)
Year of residency	1	5 (8.8)	26 (44.8)
	2	11 (19.3)	19 (32.8)
	3	10 (17.5)	13 (22.4)
	4	16 (28.1)	N.A.^a^
	5	15 (26.3)	N.A. ^a^
Frequency of encountering patients with schizophrenia	Daily	43 (75.4)	0 (0)
	Weekly	13 (22.8)	5 (8.6)
	Monthly	1 (1.8)	28 (48.3)
	Yearly	0 (0)	19 (32.8)
	> Yearly	0 (0)	6 (10.3)
Frequency of encountering patients with hypertension	Daily	28 (49.1)	53 (91.4)
	Weekly	25 (43.9)	3 (5.2)
	Monthly	4 (7.0)	2 (3.4)
	Yearly	0 (0)	0 (0)
	> Yearly	0 (0)	0 (0)
Frequency of encountering patients with diabetes mellitus	Daily	27 (47.4)	51 (87.9)
	Weekly	23 (40.4)	5 (8.6)
	Monthly	6 (10.5)	2 (3.4)
	Yearly	1 (1.8)	0 (0)
	> Yearly	0 (0)	0 (0)
Frequency of encountering patients with dyslipidemia	Daily	29 (50.9)	53 (91.4)
	Weekly	23 (40.4)	3 (5.2)
	Monthly	5 (8.8)	2 (3.4)
	Yearly	0 (0)	0 (0)
	> Yearly	0 (0)	0 (0)

^a^Family Medicine residents only have 3 years of training, while Psychiatry residents have 5 years of training

### Group differences in knowledge of schizophrenia, hypertension, diabetes mellitus, and dyslipidemia

Psychiatry residents and Family Medicine residents differed significantly on the total score of each condition. Psychiatry residents had higher scores on questions about schizophrenia versus Family Medicine residents (mean 50.70 versus 43.28, *p* < 0.001). However, Psychiatry residents scored significantly lower on questions about hypertension (mean 33.86 versus 40.98, *p* < 0.001), diabetes mellitus (mean 45.68 versus 49.79, *p* = 0.005) and dyslipidemia (mean 37.04 versus 44.31, *p* < 0.001), compared to Family Medicine residents. Table 2 shows the differences between Psychiatry residents and Family Medicine residents in terms of their knowledge of schizophrenia, hypertension, diabetes mellitus, and dyslipidemia.

**Table 2:** Differences between Psychiatry residents and Family Medicine residents in knowledge of schizophrenia, hypertension, diabetes mellitus, and dyslipidemia

Total score of questions on each condition	Mean (SD)		***P*** value
	Psychiatry resident	Family Medicine resident	
Schizophrenia	50.70 (5.16)	43.28 (7.62)	< 0.001^*^
Hypertension	33.86 (8.52)	40.98 (7.94)	< 0.001^*^
Diabetes mellitus	45.68 (7.61)	49.79 (7.61)	0.002^*^
Dyslipidemia	37.04 (7.95)	44.31 (6.34)	< 0.001^#^

Abbreviations: *SD* Standard deviation

^*^P value was calculated using Mann-Whitney U test. ^#^*P* value was calculated using T test

### Factors associated with the knowledge of Psychiatry residents

We performed multiple regression analyses to assess whether any characteristics of Psychiatry residents were associated with their knowledge of medical management of hypertension, diabetes mellitus, and dyslipidemia (Table 3). We found that receiving undergraduate medical education locally, compared to receiving undergraduate medical school overseas, was associated with better knowledge of hypertension (beta = 0.515, *p* = 0.009) and dyslipidemia (beta = 0.559, *p* = 0.005); while younger age (26–30 versus > 35 and 31–35 versus > 35) was associated with better knowledge of hypertension (beta = 1.361, *p* = 0.002 and beta = 1.225, *p* = 0.003). In other words, Psychiatry residents who graduated from local medical school appeared to have better knowledge of hypertension and dyslipidemia, while Psychiatry residents younger than 35 years tended to know more about the management of hypertension.

**Table 3:** Multiple regression analysis of factors affecting Psychiatry resident’s knowledge regarding management of hypertension, diabetes mellitus, and dyslipidemia

		Hypertension			Diabetes Mellitus			Dyslipidemia		
		Beta	***P***	95% CI	Beta	***P***	95% CI	Beta	***P***	95% CI
Gender	Male	−0.09	0.959	− 0.366, 0.347	− 0.166	0.385	− 0.547, 0.215	− 0.289	0.109	− 0.646, 0.067
	Female	Reference			Reference			Reference		
Age, years	26–30	1.361	**0.002**	0.535, 2.187	0.619	0.164	−0.263, 1.502	0.478	0.248	−0.344, 1.300
	31–35	1.225	**0.003**	0.441, 2.010	0.580	0.171	−0.258, 1.418	0.481	0.219	−0.296, 1.258
	> 35	Reference			Reference			Reference		
Medical school	Local	0.515	**0.009**	0.133, 0.898	0.203	0.324	−0.207, 0.613	0.559	**0.005**	0.181, 0.937
	Overseas	Reference			Reference			Reference		
Year of residency		−0.078	0.383	−0.257, 0.100	0.028	0.773	−0.165, 0.221	−0.168	0.064	−0.345, 0.010
Working experience in psychiatry		0.008	0.357	−0.009, 0.025	0.007	0.432	−0.011, 0.025	−0.001	0.862	−0.018, 0.015
Working experience in family medicine		−0.141	0.413	−0.484, 0.202	−0.092	0.613	−0.457, 0.273	− 0.072	0.670	− 0.411, 0.267
Frequency of encountering patients with hypertension		−0.094	0.491	−0.366, 0.178	NA	NA	NA	NA	NA	NA
Frequency of encountering patients with diabetes mellitus		NA	NA	NA	−0.013	0.916	−0.261, 0.234	NA	NA	NA
Frequency of encountering patients with dyslipidemia		NA	NA	NA	NA	NA	NA	−0.080	0.540	−0.341, 0.181

Abbrevations: *CI* Confidence Interval, *NA* Not applicable, because variable was not included in the regression analysis

### Overall opinion of Psychiatry residents towards managing schizophrenia, hypertension, diabetes mellitus, and dyslipidemia

Most Psychiatric residents (96.5%) agreed or strongly agreed that training provided to manage schizophrenia was adequate, while the majority of Family Medicine residents (62.1%) did not agree that they had adequate training to manage schizophrenia. More than half of the Psychiatry residents (61.4%) did not agree (strongly disagree or disagree) that the training provided to manage hypertension, diabetes mellitus, and dyslipidemia was adequate. A smaller proportion of Psychiatry residents did not agree that they felt confident in managing hypertension (26.3% versus 28.1% who expressed confidence), diabetes mellitus (35.1% versus 21.1% who expressed confidence), or dyslipidemia (28.1% versus 31.6% who expressed confidence). Table 4 shows the overall opinion of Psychiatry residents regarding management of schizophrenia, hypertension, diabetes mellitus, and dyslipidemia.

**Table 4:** Overall opinion of Psychiatry residents regarding schizophrenia, hypertension, diabetes mellitus, and dyslipidemia

Questions	Psychiatry residents’ responses, n (%)		
	Strongly disagree/ disagree/	Neutral	Strongly agree/ agree
I feel the training provided to manage schizophrenia was adequate	2 (3.5)	0 (0)	55 (96.5)
I feel the training provided to manage hypertension, diabetes mellitus, and dyslipidemia was adequate	35 (61.4)	19 (33.3)	3 (5.3)
I feel confident in managing patients with schizophrenia	0 (0)	0 (0)	57 (100)
I feel confident in managing patients with hypertension	15 (26.3)	26 (45.6)	16 (28.1)
I feel confident in managing patients with diabetes mellitus	20 (35.1)	25 (43.9)	12 (21.1)
I feel confident in managing patients with dyslipidemia	16 (28.1)	23 (40.4)	18 (31.6)

## Discussion

To the best of our knowledge, this is the first survey focusing on the knowledge of Psychiatry residents and Family Medicine residents regarding the medical management of schizophrenia, hypertension, diabetes mellitus, and dyslipidemia. The survey found that Psychiatry residents frequently encountered patients with hypertension, diabetes mellitus, and dyslipidemia, but felt that they did not have adequate knowledge or training in managing these medical conditions.

Most Psychiatry residents (about 90%) encountered patients with hypertension, diabetes mellitus, or dyslipidemia on a daily to weekly basis. This finding is not surprising due to the high prevalence of metabolic syndrome both in the general population and in patients with schizophrenia [10, 24, 25]. Compared to the general population, multi-episode patients with schizophrenia were found to be at increased risk for hypertension (odds ratio or OR = 1.36), low high-density lipoprotein cholesterol (OR = 2.35), hypertriglyceridemia (OR = 2.73), and diabetes mellitus (OR = 1.99) [26]. Several mechanisms may explain why patients with schizophrenia tend to have hypertension, diabetes mellitus, or dyslipidemia. Second-generation antipsychotics are strongly associated with metabolic side effects. The scientific bases of this are not yet elucidated, but some evidence suggets it may be related to the activation of sterol regulatory element-binding protein 1c (SREBP1c), the dopamine, gamma-aminobutyric acid and 5-hydroxytryptamine neurotransmission [27]. Furthermore, the increase in metabolic syndrome has also been reported in antipsychotic naive patients. It is also suggested that there may be some shared genetic risk between schizophrenia and metabolic syndrome [28]. Lastly, patients with schizophrenia tend to have a lifestyle that increases the risk for hypertension, diabetes mellitus, and dyslipidemia, including a sedentary lifestyle, poor food intake, and substance use [29]. While most Psychiatry residents encountered patients with hypertension, diabetes mellitus, or dyslipidemia frequently, nearly half of the Family Medicine residents only saw patients with schizophrenia on a monthly basis. This could explain why a large portion of Family Medicine residents did not agree that they had adequate training to manage schizophrenia.

Compared to Family Medicine residents, Psychiatry residents had significantly less knowledge of hypertension, diabetes mellitus, or dyslipidemia. When assessing knowledge of hypertension, diabetes mellitus, and dyslipidemia, we used the statements which were extracted or modified from the Singapore Ministry of Health Clinical Practice Guidelines [20–23]. As mentioned in the guidelines, they are “developed for all healthcare professionals in Singapore” [20–23]. Theoretically, Psychiatry residents should also know these guidelines. However, with increasing specialization, doctors, if not receiving continuing training, may have a sense of discomfort in managing conditions which fall outside the scope of their specialties. The use of specialty medical services has been rising, with more and more referrals between different specialties [5]. Physicians need to be aware of the disadvantages of specialization, including only focusing on the knowledge in one’s specialty and losing sight of the bigger picture [30]. Some researchers point out that a specialist treating a single chronic disease tends to focus on that disease, and may inadvertently pay less heed to other co-occurring conditions [31]. Similarly, our study suggests that Psychiatry residents are likely to focus on psychiatric disorders, such as schizophrenia, and may need updates in knowledge of some common conditions, such as hypertension, diabetes mellitus, and dyslipidemia, which they encounter frequently.

Psychiatry residents who graduated from local medical schools appeared to have a relatively better knowledge of hypertension and dyslipidemia, while Psychiatry residents younger than 35 years tended to know more about managing hypertension. The local medical education system seems to have a more comprehensive approach [32]. In Singapore, there are three medical schools: Yong Loo Lin School of Medicine, National University of Singapore (NUS Medicine), Duke-NUS Medical School, and Lee Kong Chian School of Medicine. This creates an environment of friendly competition in Singapore medical education [32]. The good quality of the local medical education system may help Psychiatry residents to have better knowledge of hypertension and dyslipidemia. On the other hand, younger Psychiatry residents, compared to those who are 35 years old or above, are more likely to remember about what they have just learnt in medical school, hence they know more about the management of hypertension.

More than half of the Psychiatry residents (61.4%) did not agree that the training provided to manage hypertension, diabetes mellitus, and dyslipidemia was adequate. Compared to hypertension and dyslipidemia, a slightly greater proportion of Psychiatry residents did not agree that they felt confident in managing diabetes mellitus. There are multiple factors that may influence these findings which may be related to practice and program considerations. Practice factors include real-world practice culture in the management of comorbid medical issues of varying severity in patients with schizophrenia by the same treating psychiatrist, thresholds for referring such patients with comorbid hypertension, diabetes, dyslipidemia to medical colleagues, opportunities to manage in the context of clinical site policies, de-skilling or disempowerment over time. Further studies are needed to tease out the specific issues underlying these observations.

This study highlights Psychiatry residents’ sense of discomfort in managing comorbid medical conditions in patients with schizophrenia. Possible considerations include continuing emphasis on awareness and management of these common medical conditions within the compulsory Internal Medicine rotations within the residency training, updates on management of these conditions over time as part of lifelong continual learning and refining residency selection criteria into the training program. The selection process for entry also does not require the potential resident to have completed a posting in Family Medicine prior to joining the psychiatry residency. In the late 2000s, Singapore shifted its postgraduate training program from the British basic and advanced specialty training system to the American residency system [32, 33]. Interestingly, in the United States, there is a combined family medicine-psychiatry training program [34–37]. Residents in the combined family medicine-psychiatry training program report high levels of satisfaction and plan to practice in both specialties [34]. Given the potential benefits to patients from the integrated care, another consideration is to foster closer collaboration between psychiatry residents and family medicine practitioners within the clinical setting in the holistic management of patients with psychiatric conditions.

There are a few limitations to our study. First, the survey questionnaire used in this study is not a validated tool. However, it was developed based on local clinical practice guidelines and was discussed with domain experts. Second, although we aimed to recruit all Psychiatry residents into the study, only 81.4% responded to the survey. Third, the study was conducted in the local setting; the medical training system in other countries may be different, and hence the results may not be applicable to them.

## Conclusions

This study raises the awareness of Psychiatry residents’ sense of discomfort in managing hypertension, diabetes mellitus, or dyslipidemia, though they encounter patients with these conditions frequently. Future studies are warranted to examine the systemic (such as practice patterns, policies, culture, opportunities regarding the management of comorbid medical conditions in patients with schizophrenia) and local (such as training curriculum) factors which may influence our study findings in order to better refine residency selection criteria and training program to impact real-world practice.

## Supplementary Information

**Additional file 1.** Knowledge of Psychiatry and Family Medicine residents regarding management of schizophrenia, hypertension, diabetes mellitus, and dyslipidemia.

## Data Availability

The datasets used and/or analyzed during the current study are available from the corresponding author on reasonable request.

## Abbreviations

ACGME-I: Accreditation Council for Graduate Medical Education-International
NHG: National Healthcare Group
COVID-19: Coronavirus Disease 2019
SD: Standard deviation
CI: Confidence interval
NA: Not applicable
OR: Odds ratio
SREBP1c: Sterol regulatory element-binding protein 1c
NUS Medicine: Yong Loo Lin School of Medicine, National University of Singapore
