# Supplementary material for: Perceived knowledge of psychiatry and family medicine residents regarding medical management of schizophrenia, hypertension, diabetes mellitus, and dyslipidemia: opportunities to refine the residency training
Source: BMC Med Educ. 2021 Apr 22;21:232. doi: 10.1186/s12909-021-02658-z (PMC8061461; doi:10.1186/s12909-021-02658-z)
Supplement: Supplementary file 1 — Additional file 1. Knowledge of Psychiatry and Family Medicine residents regarding management of schizophrenia, hypertension, diabetes mellitus, and dyslipidemia. [file 12909_2021_2658_MOESM1_ESM.pdf]

Perceived knowledge of Psychiatry and Family Medicine residents regarding medical management of schizophrenia, hypertension, diabetes mellitus, and dyslipidemia

Demographic information (please circle your response)						
Gender		Male		Female		
Age		20-25	26-30	31-35	36-40	>40
Medical school		Local		Overseas		
Residency program		Psychiatry		Family Medicine		
Year of residency		1	2	3	4	5 or above
Have you had working experience in psychiatry before residency program						
		Yes		No		
If yes, approximately how many months			( ) months			
Have you had working experience in family medicine before residency program						
		Yes		No		
If yes, approximately how many months			( ) months			
How frequent do you encounter patients with the following conditions						
Schizophrenia		daily	weekly	monthly	yearly	>yearly
Hypertension		daily	weekly	monthly	yearly	>yearly
Dyslipidemia		daily	weekly	monthly	yearly	>yearly
Diabetes mellitus		daily	weekly	monthly	yearly	>yearly

Please circle the preferred numeric response to each statement							
	Management	Survey scale:<br>1=strongly disagree;<br>2=disagree;<br>3=neutral; 4=agree;<br>5=strongly agree<br>0=don't know					
**Schizophrenia**							
1	People newly diagnosed with schizophrenia should be offered oral antipsychotic medication.	0	1	2	3	4	5
2	If there is inadequate response by 4-6 weeks or if patient develops intolerable side effects, the medication should be reviewed and another typical or atypical antipsychotic should be used.	0	1	2	3	4	5
3	Oral antipsychotics should be used as first-line treatment for patients with an acute relapse of schizophrenia.	0	1	2	3	4	5
4	For maintenance therapy, antipsychotic dose should be reduced gradually to the lowest possible effective dose, which should not be lower than half of the effective dose	0	1	2	3	4	5

	during the acute phase.						
5	Clozapine should be offered to patients whose illness has not responded adequately to treatment despite the sequential use of adequate doses and duration of at least two different antipsychotics.	0	1	2	3	4	5
6	Combination of antipsychotics is not recommended except during transitional periods when patients are being switched from one antipsychotic to another, or when used for clozapine augmentation.	0	1	2	3	4	5
7	Electroconvulsive therapy can be prescribed as first-line treatment or monotherapy in schizophrenia.	0	1	2	3	4	5
8	Antidepressants should not be considered when depressive symptoms emerge during the stable phase of schizophrenia.	0	1	2	3	4	5
9	Long-acting depot antipsychotics should not be used for acute episodes because it may take 3-6 months for the medication to reach a stable steady state.	0	1	2	3	4	5
10	Patients receiving atypical antipsychotics should be regularly monitored for metabolic side effects.	0	1	2	3	4	5
11	For all patients with clozapine, clinician should have their full blood count monitored weekly for the first 18 weeks and monthly thereafter.	0	1	2	3	4	5
12	Cognitive remediation is not effective among people with schizophrenia.	0	1	2	3	4	5
**Hypertension**							
1	If an adequate dose of the first drug used demonstrated limited response or was poorly tolerated, change to a different drug class instead of increasing the dose of the first drug.	0	1	2	3	4	5
2	A diuretic is recommended as initial treatment in patients with uncomplicated hypertension who are at risk for diabetes mellitus.	0	1	2	3	4	5
3	For patients with type 2 diabetes mellitus who have hypertension, an acceptable treatment target blood pressure (BP) is below 140/80 mmHg.	0	1	2	3	4	5
4	Beta-blockers, calcium-channel blockers and thiazides are not recommended for second-line therapy in diabetic nephropathy.	0	1	2	3	4	5
5	Advise patient to restrict salt intake to 5g to 6g per day.	0	1	2	3	4	5
6	Can allow moderate alcohol consumption to no more than two standard drinks per day for men, and no more than one standard drink per day for women.	0	1	2	3	4	5
7	Aggressive rate of lowering of BP in pregnant women with chronic hypertension is recommended.	0	1	2	3	4	5
8	Use beta-blockers with caution in patients at risk of	0	1	2	3	4	5

	developing diabetes mellitus, as it raises blood glucose concentrations.						
9	Methyldopa, labetalol and nifedipine are not recommended for use during breastfeeding postpartum.	0	1	2	3	4	5
10	Renal sympathetic denervation can be offered for routine treatment of resistance hypertension.	0	1	2	3	4	5
11	In elderly patients with isolated systolic hypertension, calcium-channel blockers and diuretics are not recommended.	0	1	2	3	4	5
12	In elderly hypertensive patients whose systolic BP is 160 mmHg or higher, the BP should be reduced to below 150/90 mmHg.	0	1	2	3	4	5
**Diabetes Mellitus**							
1	The HbA1c target for most non-pregnant adults with type 1 or type 2 diabetes should be ≤7.0% or ≤53 mmol/mol.	0	1	2	3	4	5
2	Metformin is usually not considered first-line pharmacotherapy.	0	1	2	3	4	5
3	For type 2 diabetes, two or more oral agents, or insulin therapy, either alone or in combination with oral agents, may be required.	0	1	2	3	4	5
4	All patients with type1 diabetes must receive insulin.	0	1	2	3	4	5
5	Screening of diabetes mellitus should be considered in adults of any age who have one or more risk factors for diabetes. In those without risk factors, testing should begin at 40 years.	0	1	2	3	4	5
6	Self-monitoring of urine glucose is recommended for monitoring of glycemic status.	0	1	2	3	4	5
7	Metformin is usually contraindicated in the presence of severe renal or hepatic insufficiency.	0	1	2	3	4	5
8	All patients diagnosed with diabetes require regular visual acuity assessment and eye examinations.	0	1	2	3	4	5
9	Breastfeeding is not recommended for infants of women with diabetes.	0	1	2	3	4	5
10	It is not recommended to consider low-dose aspirin in diabetic individuals with a history of vascular disease.	0	1	2	3	4	5
11	Hyperlipidemia should be treated to retard diabetic retinopathy.	0	1	2	3	4	5
12	Laser therapy should be offered to patients with proliferative diabetic retinopathy and diabetic macular edema.	0	1	2	3	4	5
**Dyslipidemia**							
1	Clinician should routinely screen men and women aged 40 years and older for lipid disorders.	0	1	2	3	4	5

2	Due to risk of myopathy and rhabdomyolysis, high dosage of statins should be prescribed with caution.	0	1	2	3	4	5
3	Elevation in the levels of serum transaminases above three times the upper limit of the normal range is an indication to stop statins.	0	1	2	3	4	5
4	Elevation of serum creatine kinase greater than 5-10 times the upper limit of the normal range, when associated with muscle pain, is an indicate to stop statins.	0	1	2	3	4	5
5	Statins are the first line drug for both hypercholesterolemia and mixed hyperlipidemia when pharmacotherapy is indicated.	0	1	2	3	4	5
6	Ezetimibe cannot be used as an add-on drug in association with statins.	0	1	2	3	4	5
7	Statins can be used in women who are pregnant.	0	1	2	3	4	5
8	Fibrates cannot be used in patients with chronic kidney disease in stage 1-3.	0	1	2	3	4	5
9	Screening of all first degree relatives of diagnosed familial hypercholesterolemia patients is recommended.	0	1	2	3	4	5
10	Resins cannot be used in children with dyslipidemia.	0	1	2	3	4	5
11	In severe hypertriglyceridemia, where fibrates alone may not adequately lower the markedly elevated triglyceride (TG) levels, omega 3 fish oils can be added.	0	1	2	3	4	5
12	Statin cannot be used in children with dyslipidemia.	0	1	2	3	4	5
**Training**							
1	I feel the training provided to manage schizophrenia was adequate.	1	2	3	4	5	
2	I feel the training provided to manage hypertension, diabetes and dyslipidemia was adequate.	1	2	3	4	5	
**Overall**							
1	I feel confident in managing patients with schizophrenia.	1	2	3	4	5	
2	I feel confident in managing patients with hypertension.	1	2	3	4	5	
3	I feel confident in managing patients with diabetes.	1	2	3	4	5	
4	I feel confident in managing patients with dyslipidemia.	1	2	3	4	5	
5	I feel confident in managing patients with both schizophrenia and metabolic syndrome (hypertension, diabetes and/or dyslipidemia).	1	2	3	4	5	
